# Parliament and Publications

**Published:** 1910-11-26

**Authors:** 


### Parliament and Publications.

### RAT PLAGUE IN EAST SUFFOLK.

In the House of Lords on November 22, Lord Allendale detailed the steps that had been taken by the Local Government Board with a view to the extermination of rats in certain districts. His Lordship said that up till November 21 only six districts were known to have rat plague in East Suffolk, one in West Suffolk, and one in Essex.

### PLAGUE VACCINE.

Mr. Lionel de Rothschild intends to ask the President of the Local Government Board whether, in the event of plague epidemic breaking out in England, there is a sufficient supply of preventative and curative vaccine for the use of medical men in this country; and, if not, whether he would consider the advisability of making arrangements to have the same in readiness.

### ANTI-TOXIN AND THE DEATH RATE FROM DIPHTHERIA.

Mr. Chancellor has given notice to ask the President of the Local Government Board what is the authority by which his department was empowered to issue the recent order to local authorities suggesting the supply of anti-toxin to private medical practitioners out of the public rates and the employment of special medical aid for its administration without reference to Parliament. He further intends to ask whether the President of the Board is aware that the Registrar-General's returns report that during the last fifteen years, since anti-toxin has been introduced, the death-rate from diphtheria to the living population of this country has increased by 25 per cent. above that of fifteen years prior to its introduction; and that in the tables supplied by the Metropolitan Asylums Board's reports for the past fifteen years the cases of diphtheria treated without anti-toxin have declined double as much as those cases which were treated with it.

### THE POLITICAL SITUATION.

In the House of Commons on Friday of last week, the Prime Minister announced that the Government had advised the Crown to dissolve Parliament at the earliest possible moment—namely, November 28. In the meantime the House will proceed to a conclusion with the essential parts of the Finance Bill. By an immediate issue of the writs the General Election may conclude a few days before Christmas. It does not appear from the lists of selected candidates that an appreciably greater number of medical practitioners will contest seats than at the last election. Among the candidates for London constituencies are Sir William Collins, Dr. Christopher Addison, Dr. R. Moon, and Dr. J. E. Molson. At the last election Sir W. Collins was returned as the Liberal member for St. Pancras W. by a narrow majority; Dr. Addison, the sitting member for Hoxton, gained a seat for the Liberal party. Both these candidates will seek re-election in the same constituencies. Dr. R. Moon will again contest Marylebone E. in the Liberal interest, a constituency in which he was unsuccessful at the last election. Dr. J. E. Molson has been selected as Conservative candidate for Bethnal Green N.E., a seat which is at present held by a Liberal, whose majority in January was 1,407. As at the last election, Mr. W. S. Glyn-Jones, the Pharmaceutical Society's Parliamentary Secretary, will contest Stepney, a constituency in which he was beaten by the Conservative candidate. Mr. Glyn-Jones was for many years in business as a chemist and druggist, but is now in practice as a barrister.

